# Reaction on Twitter to a Cluster of Perinatal Deaths: A Mixed Method Study

**DOI:** 10.2196/publichealth.5333

**Published:** 2016-07-27

**Authors:** Sarah Meaney, Leanne Cussen, Richard A Greene, Keelin O'Donoghue

**Affiliations:** ^1^ National Perinatal Epidemiology Centre Obstetrics and Gynaecology University College Cork Cork Ireland; ^2^ Pregnancy Loss Research Group Department of Obstetrics and Gynaecology University College Cork Cork Ireland

**Keywords:** social media, health care services, maternity, perinatal death, Twitter, infodemiology, infoveillance

## Abstract

**Background:**

Participation in social networking sites is commonplace and the micro-blogging site Twitter can be considered a platform for the rapid broadcasting of news stories.

**Objective:**

The aim of this study was to explore the Twitter status updates and subsequent responses relating to a number of perinatal deaths which occurred in a small maternity unit in Ireland.

**Methods:**

An analysis of Twitter status updates, over a two month period from January to March 2014, was undertaken to identify the key themes arising in relation to the perinatal deaths.

**Results:**

Our search identified 3577 tweets relating to the reported perinatal deaths. At the height of the controversy, Twitter updates generated skepticism in relation to the management of not only of the unit in question, which was branded as unsafe, but also the governance of the entire Irish maternity service. Themes of concern and uncertainty arose whereby the professional motives of the obstetric community and staffing levels in the maternity services were called into question.

**Conclusions:**

Twitter activity provides a useful insight into attitudes towards health-related events. The role of the media in influencing opinion is well-documented and this study underscores the challenges that clinicians face in light of an obstetric media scandal. Further study to identify how the obstetric community could develop tools to utilize Twitter to disseminate valid health information could be beneficial.

## Introduction

During pregnancy women are invested in seeking out a considerable amount of information in relation to pregnancy and the services that are available to them [1]. To date the provision of pregnancy-related information during the antenatal period has been through more traditional media [1]. These, including leaflets, magazines and advertisements, channel the information directly from source to target audience [2]. Thus, women are limited to the passive viewing of pregnancy-related content, which has been created for them [3].

Rapid development in Web-based technologies has seen a shift in how women now access pregnancy-related information. A recent study has shown that 95% of pregnant women in Ireland use the Internet for pregnancy information [1]. The transition from more traditional to digitally-based media may be related to two issues: (1), women of child-bearing age in developed countries have access to a wide array of technologies including personal computers, laptops, tablets and smartphones; (2) women may have more confidence in the information that they receive online given how Stapleton et al found that traditional materials such as leaflets or books were considered not only limited and biased but that the information provided was considered dated [4].

Health care providers and policymakers need to address the information needs of pregnant women [5]. The benefit of Internet-based technologies for women is that they are no longer passive users. The development of these technologies ensures that information provision is consumer-centered whereby not only users are encouraged to interact with others but also to create and share content through multiple digital channels [6]. Bernhardt et al outline how this revolution in communication has already affected health care as a new generation of e-patients has emerged [3]. Digital media has empowered these health care consumers, allowing them to be even more engaged in their care and in turn to influence their current and future service provision [3].

The expansion of social network platforms in particular has driven these developments. Participation in social networking websites such as the micro-blogging site Twitter is now commonplace. It is reported that 38 million adults in the United Kingdom access the Internet daily, with over half of the population participating in social networking [7]. A national cross-sectional survey from 2015 indicated that 26% of the Irish population use Twitter, with one in three of those people using Twitter daily [8]. However, a tweet extends far beyond the individual status as it includes its audience; those who may read the tweet, retweet and/or reply [9]. As any status update can be seen quickly by a very large audience [10], Twitter must be considered a platform for rapid and immediate communication. These sites allow individuals to share their thoughts on the information they are currently consuming [10]. Such rapid communications among these new e-patients are very likely to be influential in how people consume information in relation to health care services [10]. Health care consumers now have a new digital space where they can discuss information that is provided to them and evaluate their health care services as they experience it, either while attending a clinic or while watching a TV broadcast [10]. This phenomenon has already been observed in the United Kingdom where reforms in the National Health Service have been influenced by social commentary on Twitter [11].

Social media is transforming health-related research [12]. Studies have been undertaken from a wide range of disciplines, from epidemiology where real time responses to pandemics are analyzed [13] to the behavioral sciences where the way patients consume online health-related information is examined [3,14]. The manner in which women both access and respond to pregnancy-related information needs to be evaluated as it is likely to be influential in relation to women’s decision making [4].

Reports in the media related to pregnancy and birth are common and these reports are often emotive [15]. Such reporting may have a detrimental impact as it can potentially misinform pregnant women and may possibly result in confusion and anxiety [15]. How news is communicated has altered dramatically as websites, social media, and 24-hour rolling broadcasts have seen rapid growth and increased popularity [15]. Since 2012, there has been considerable national and international media coverage reporting a number of adverse incidents within the Irish maternity services, which in turn have resulted in a number of independent enquiries into the services. The media coverage of these adverse incidents stimulated much debate, including discussion in relation to a cluster of perinatal deaths in Midland Regional Hospital, Portlaoise in 2014. Although there is ongoing research on the causes of perinatal death, which aims to reduce its prevalence, there is still persistent stigma associated with perinatal death [16]. Stillbirth, in particular, is often referred to as a silent loss [16]. Thus, our study aimed to explore the reaction on Twitter to the perinatal deaths in order to gain insight into the understanding and perception of perinatal death in the Republic of Ireland.

## Methods

### Setting

In 2014, the Irish Central Statistics Office estimated that the Irish population was 4,593,100 [17]. In Ireland, the Maternity and Infant Care Scheme grants women ordinarily resident in Ireland access to free maternity services. This public service is provided by both a general practitioner and a maternity health care provider. The majority of births occur in one of the 19 obstetric led units in Ireland [18]. Ireland has the highest birth rate in Europe with 15.6 births per 1000 population [19]. In 2014, there were 67,462 births of which 330 were stillbirths; defined here as an infant born with no sign of life weighing 500 grams or more and/or having a gestational age of 24 weeks or more [20].

Raidió Teilifís Éireann (RTE) is an Irish television broadcaster. As a national public-service media organization the service which it provides is free to air [21]. RTE’s flagship current affairs program is Primetime. In 2015 when this study was conducted, RTE had 205,000 followers on Twitter and the Primetime account had 69,000 followers. On January 30, 2014 RTE aired a program entitled “Fatal Failures” on Primetime. The program was concerned with a cluster of perinatal deaths that occurred in a maternity hospital located in the Midlands in the Republic of Ireland. Following this program the Health Minister, James Reilly, requested the Chief Medical Officer to prepare a report on the issues identified in the program. The Chief Medical Officer published the report on February 28, 2014 [22]. The Health Minister also requested the Health Information Quality Authority, an independent authority who is responsible for assessing quality and safety of health care services, to undertake a review of the hospital in question.

### Design

As this study was an observational study of Twitter status updates in relation to the reported perinatal death, a mixed methods approach was adopted. By utilizing a mixed methods design the study was able to benefit from analyzing the data both quantitatively and qualitatively. For the purpose of this study the data were initially quantitatively analyzed in order to assess the frequency of status updates, the demographic profiles of users, and to ascertain the potential reach of the status updates. Secondly, qualitative analysis was employed to generate themes from the content that Twitter users shared publically.

### Search Strategy

Status updates in English were manually searched using the Twitter search function on its website. A search was undertaken on all public status updates from January 29, 2014 to March 31, 2014 relating to the perinatal deaths in the Republic of Ireland. The two month period was chosen as research indicates that public interest spike around the time of the event and decline rapidly thereafter [23]. This time frame was chosen to allow for the examination of the immediate response to the reporting of the perinatal deaths and the subsequent reports published in relation to the perinatal deaths.

Seven searches were conducted independently by two researchers (SM and LC) which included: “fatal failures” (the name of the episode regarding the perinatal deaths), “rtept” (the current affairs program which aired the episode), “death of a baby”, “maternity”, “stillbirth”, “perinatal death”, and finally we searched the name of the hospital where the deaths occurred. Initially the searches were limited to using hashtags (#) which are useful to search for content on Twitter as they group messages on a specific event together. However, we found that this limited the search. Therefore, we chose to proceed using the terms alone for the search process as it produced more results, including the hashtags related to the perinatal deaths. All data which were extracted from each of the searches were stored in Microsoft Excel. Each tweet was then reviewed and assessed for inclusion in the study.

Once these searches were complete, any demographic information available was collected from the users’ public profiles. This included their biography, their location, and the number of people who were following the user at the time of data collection in 2015.

Only data which were publically available were collected and no attempts were made to contact any individual; therefore, no ethical approval was sought for this study. Despite these data being available to public, there is still an onus to ensure that ethical standards are met. Therefore, in line with other similar studies [14,24,25] personal identity information, including individuals’ Twitter usernames, have been removed from the example tweets presented below.

### Analysis

Both quantitative and qualitative methods were utilized for this study. First, in order to determine the volume of social media communication in relation to the perinatal deaths, descriptive statistics of all tweets, retweets and replies were calculated. If a user’s biography was available, this was coded by the researchers (SM and LC) in order to categorize the demographic profiles of the users. The biography on Twitter is limited to 160 characters and is the user’s self-description. Therefore, the demographic data reported here is self-identified by the user. Consequently, users were broadly grouped into the following categories which are reported here: parent, media outlet, media personnel, politics, and health. These categories were not considered mutually exclusive whereby, for example, a user may describe themselves as a “midwife and mother of two children” and therefore, would be considered both a parent and a health care professional. In order to determine the potential reach of the status updates, the number of people that were following the user was collected.

Given that this is an observational study, a qualitative methodology which is more descriptive rather than interpretative was chosen. Consequently, a thematic analysis of the text within the Twitter status updates was then performed electronically using Nvivo 10 software (QSR International Pty Ltd, Doncaster, Australia). The analytic process, as outlined by Braun and Clarke (2006), involves familiarization with the data whereby the researchers read and re-read each tweet which was then coded individually [26]. These preliminary codes were reviewed and similar individual codes were identified and grouped together as categories. The final themes were then agreed by grouping related categories together.

## Results

### Quantitative Results

Over the two month period from January 29, 2014 to March 31, 2014, 3577 Twitter status updates from 1276 profiles relating to the perinatal deaths in Midland Regional Hospital, Portlaoise were identified. Of these status updates 45.15% (1615/3577) were tweets, 38.92% (1392/3577) were retweets, and 15.94% (570/3577) were replies.

**Figure 1:**
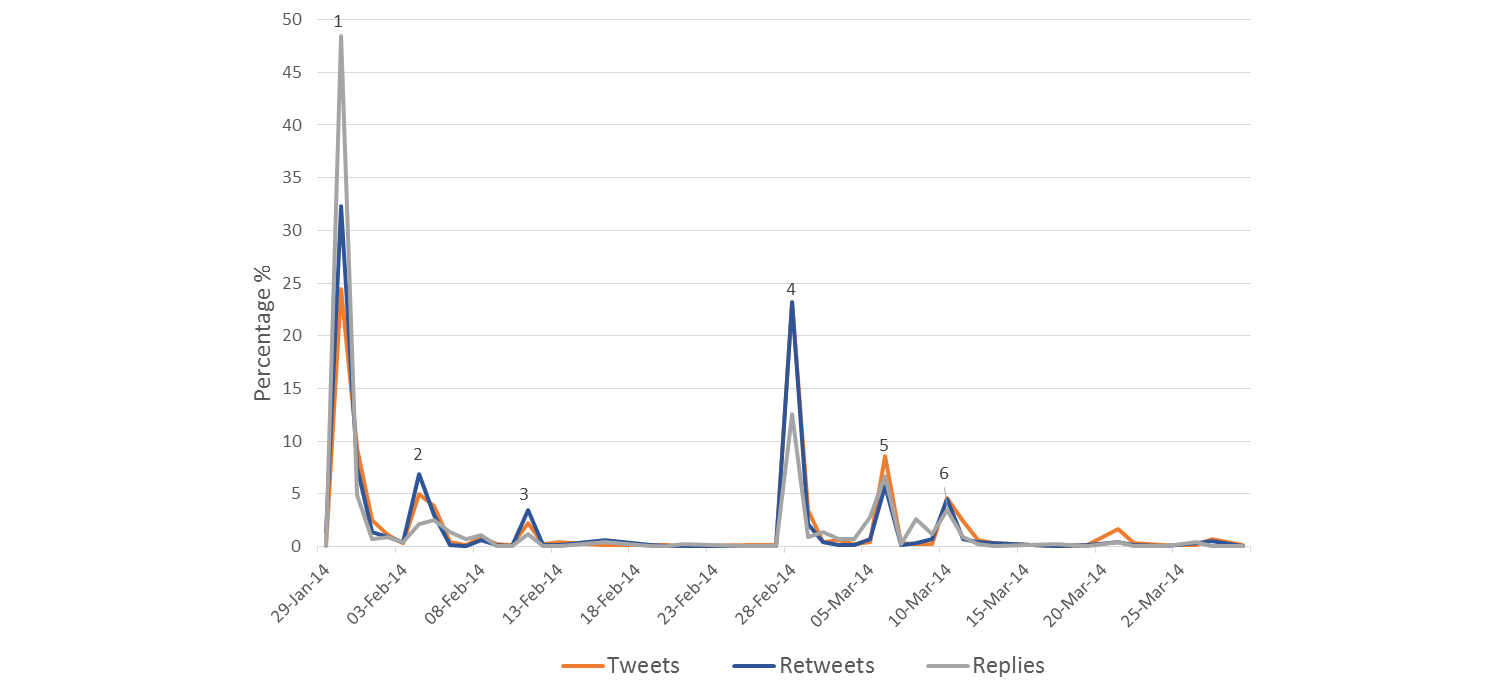
Frequency of status updates by date: (1) Current affairs television program Primetime air the episode entitled Fatal Failures relating to a cluster of perinatal deaths in Portlaoise hospital in the Republic of Ireland; (2) Minster for Health makes an emotional statement after meeting the families who had a perinatal death and announces the investigation by the Chief Medical Officer; (3) A mother releases a statement that she was only made aware that an investigation was undertaken on the perinatal death as a result of the Primetime program; (4) Findings from the Chief Medical Officer’s report are published; (5) It is confirmed that an independent investigation of the hospital will be undertaken by the Health Information Quality Authority; (6)The Health Service Executive confirm that a perinatal death occurred in Portlaoise hospital on March 8, 2014.

As illustrated in Figure 1, around 39.84% (1425/3577) of status updates were posted between the 29^th^and 31^st^of January coinciding the airing of the current affairs program which investigated the perinatal deaths. Almost half of all status updates which were replies were posted on the 30^th^of January (48.4%; 276/570). The second largest peak of status updates (21.5%; 770/3577) related to the publication of the findings from the Chief Medical Officer’s report.

The individual profiles of those who posted status updates were analyzed to discern demographic characteristics. Of the 1276 profiles, a biography was available for 1139 (89.3%). Profiles indicated that those who self-identified as being involved in media, health care and/or politics accounted for almost two thirds of status updates (62.87%; 2249/3577). More than 1 in 10 status updates were by those who self-identified as a parent (11.85%; 424/3577). Table 1 outlines the distribution of status updates among these groups. Profiles which identified the user as either a media outlet or personnel working for a media outlet accounted for over one third (36.82%; 1317/3577) of status updates. Of note, media outlets were more likely to create content; whereby the majority (83.3%) of their updates were tweets compared to other users. Parents and those involved in health care were more likely to question or discuss content with 29.0% and 24.2% of their updates being replies.

**Table 1:** Sample characteristics.

	All status updates (n=3577)	Media outlets (n=540)	Media personnel (n=777)	Health care professional (n=467)	Politics (n=465)	Parent (n=424)
Tweet n (%)	1615 (45.20)	450 (83.3)	364 (46.8)	178 (38.1)	179 (38.55)	133 (31.4)
Retweet n (%)	1392 (38.92)	90 (16.7)	310 (39.9)	176 (37.7)	202 (43.4)	168 (39.6)
Reply n (%)	570 (15.94)	0 (0)	103 (13.3)	113 (24.2)	84 (18.1)	123 (29.0)
Mean number of followers	11,709	59,437	5667	1902	3663	1138

Of the profiles related to health care (n=111), almost one third (29.7%) were from a diverse range of support organizations and online health care businesses, such as those who provide health, fitness, and nutrition services online. One in five identified as either a midwife or a nurse. Fourteen percent identified as a medical doctor or consultant; however, none were from the field of obstetrics and gynaecology. Of the political profiles (n=122), one quarter identified themselves as a political figure in Ireland such as a Member of the Irish Parliament or a local Councillor. One in six profiles saw individuals identify themselves as activists (16.4%).

**Figure 2:**
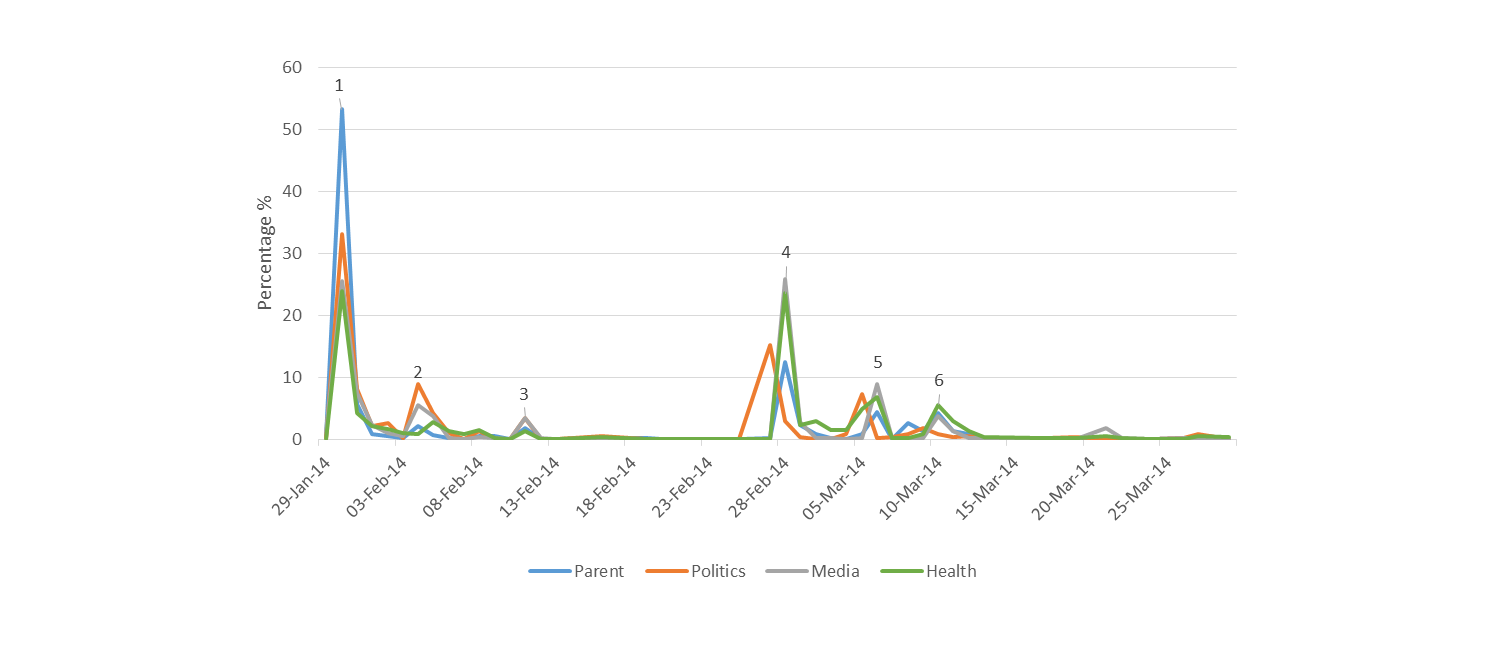
Frequency of status updates by date and user: (1) Current affairs television program Primetime air the episode entitled Fatal Failures relating to a cluster of perinatal deaths in Portlaoise hospital in the Republic of Ireland; (2) Minster for Health makes an emotional statement after meeting the families who had a perinatal death and announces the investigation by the Chief Medical Officer; (3) A mother releases a statement that she was only made aware that an investigation was undertaken on the perinatal death as a result of the Primetime program; (4) Findings from the Chief Medical Officer’s report are published; (5) It is confirmed that an independent investigation of the hospital will be undertaken by the Health Information Quality Authority; (6) The Health Service Executive confirm that a perinatal death occurred in Portlaoise hospital on March 8, 2014.

Over half of the status updates (53.3%; 226/424) posted by parents occurred on January 30^th^(Figure 2). One of the largest volumes of status updates, 13.2% (62/465), by those involved in politics occurred when the Minster for Health made an emotional statement while announcing a hospital investigation was to be undertaken. Almost one quarter (23.6%; 110/465) of status updates by those involved in health care occurred when the findings from the Chief Medical Officer’s report were published.

### Qualitative Results

#### Themes

**Figure 3:**
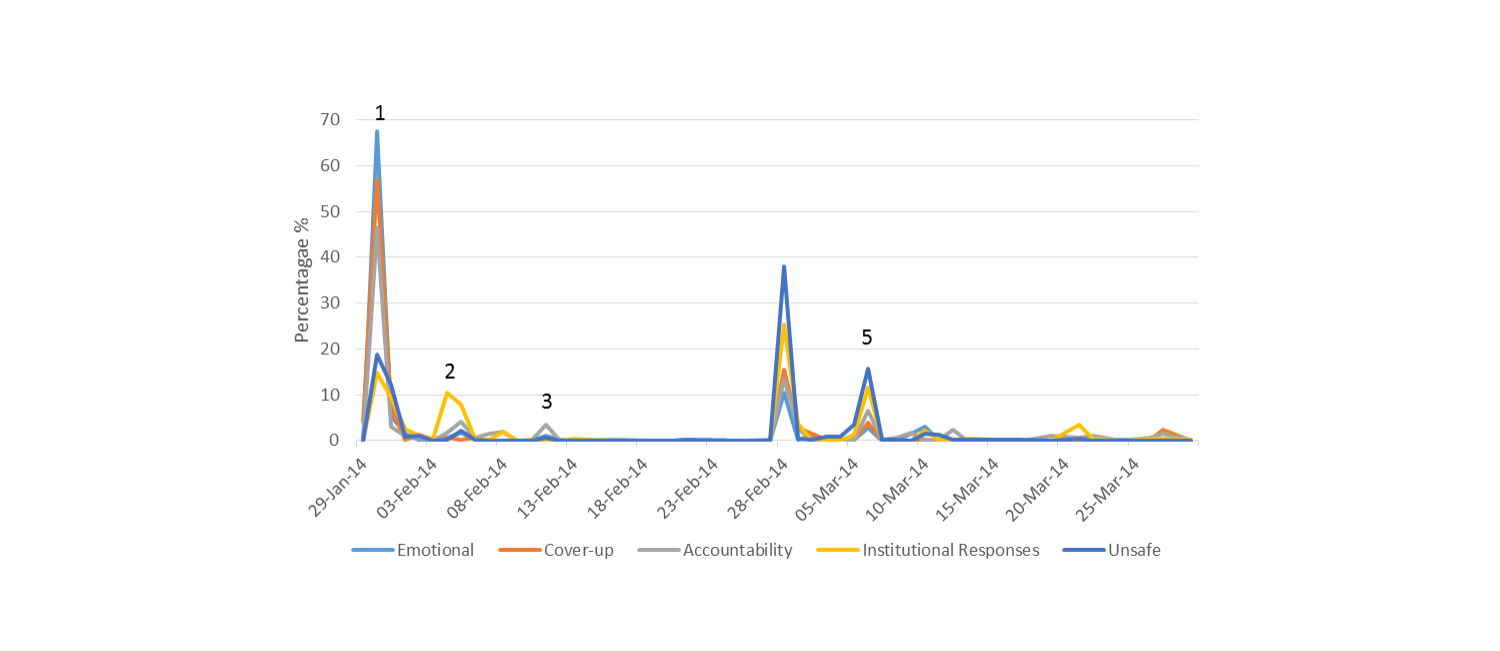
Frequency of status updates by date and theme: (1) Current affairs television program Primetime air the episode entitled Fatal Failures relating to a cluster of perinatal deaths in Portlaoise hospital in the Republic of Ireland; (2) Minster for Health makes an emotional statement after meeting the families who had a perinatal death and announces the investigation by the Chief Medical Officer; (3) A mother releases a statement that she was only made aware that an investigation was undertaken on the perinatal death as a result of the Primetime program; (4) Findings from the Chief Medical Officer’s report are published; (5) It is confirmed that an independent investigation of the hospital will be undertaken by the Health Information Quality Authority; (6) The Health Service Executive confirm that a perinatal death occurred in Portlaoise hospital on March 8, 2014.

Qualitative analysis of the tweets resulted in the identification of five key themes: emotional reactions, cover-ups, accountability and governance, institutional responses, and unsafe maternity services (Figure 3).

#### Emotional Reactions

The majority of tweets, including retweets and replies, in this theme were characterized as negative responses where anger, distress, and upset were communicated. As the current affairs program “Fatal Failures” aired, tweets indicated that the viewers of the episode were shocked and upset by what was reported. A number of status updates indicated the frightening and distressing nature of the program (Textbox 1).

Tweeters sympathized with the families for the perinatal loss they had experienced. The tweets reveal how individuals expressed empathy for these parents as they tried to comprehend the loss experienced, thankful that they themselves had not experienced such tragedy (Textbox 1).

**Examples of tweets showing emotional reactions.**

“Frightening & Distressing”

That was so incredibly sad #rtept. My son, nieces & nephews were born in Portlaoise &I'll go to bed tonight counting my blessings.

Heartbreaking and chilling at the same time- has unsettled me #expectingno3 #rtept

So terrible looking at these beautiful babies that never got a chance to live their lives. Very distressing. #rtept

“Empathy for Parents”

This is so upsetting. Feeling blessed that my little baby was delivered safely in Portlaoise. My heart goes out to those not as lucky #rtept

Absolutely shocking #rtept Report Fatal Failures, heart goes out to families, should never have happened & should never happen again!

Just bawled my eyes out all the way through @RTE_PrimeTime My heart goes out to those families who lost their babies unnecessarily #rtept

My heart goes out to the mothers and fathers featured on #rtept #primetime I don't think I'd have the strength if I was in their shoes

@RTE_PrimeTime fantastic report, impossible viewing, inspiring families, cruel cruel system. Congrats. Hard not to be angry.

“Shock & Anger”

Can only imagine the torture of the uncertainty, questions, what ifs, maybes, if onlys these parents must have gone through for years #rtept

@RTE_PrimeTime absolutely shocking behavior. Hard to watch.

Cannot believe what they did at Portlaoise hospital, an utter disgrace to the medical profession. #Sickening

Heartbreaking watching #rtept Not good enough #HSE Shame on the hospital. My thoughts are with those brave families. Devastated lives.

Disgusted by the HSE and Portlaoise Hospital. Incompetent callous and cowardly #rtept

The shock and anger expressed on Twitter were aggravated by the portrayal of the hospital staff and management. The fundamental values of care and compassion, which are normally attributed to those who provide health-related services, were at odds with the televised representation of those who were responsible for the care of the families who had experienced perinatal loss (Textbox 1).

#### Cover-ups

Tweeters indicated that they believed the hospital management’s priority was to cover up the events surrounding the perinatal deaths rather than focusing on the appropriate care for the patients (Textbox 2).

**Examples of tweets showing cover-ups and frustration with both the Irish Government and the Health Service Executive.**

“Cover-ups”

#rtept on neonatal deaths horrific viewing #HSE comes out poorly obstructing info to families, spokesman dodging issues, evasive.

#rtept I find the cover-up so much more distressing than the actual deaths

It was appalling for all but the mother who was left believing that something she did may have caused the death was just cruel

How do they sleep at night knowing they've sent somebody home not knowing why they're really leaving hospital without their baby. #rtept

“Frustration”

Are we to assume reports were made available only because #rtept were investigating?

Reilly seeks report on baby deaths…surely our Health Minister doesn't need a TV docu to prompt an investigation

#rtept shows again & again how reports are published & never implemented in our hospitals so women & babies die

We never learn! Barely a word about the HIQA #Savita report recommendations. Where is the sense of urgency about improving standards. #rtept

HSE guy - I regret “”IF“” any actions....... The usual PR speak. Maddening #rtept

Independent Hiqa inquiry into baby deaths in #Portlaoise hospital is essential. No point in HSE investigating itself. Families deserve truth

The Irish national broadcaster, and the journalists involved in the investigation, were praised for their role in informing the public firstly of the occurrence of the perinatal deaths but also of the suppression of information by the hospital.

#rtept Pubic Service Broadcasting at its best tonight! Harrowing stories of avoidable infant deaths at Portlaoise maternity hospital.

Common theme in Ireland on internal investigations, first instinct is to cover up, obstruct and frustrate. #rtept

Midwives in Portlaoise knew trouble was brewing and they were ignored. Maybe social media is the way forward so the public is informed.

Tweets indicated frustration with both the Irish Government and the Health Service Executive, whereby their actions were seen as reactive and defensive (Textbox 2). It was suspected that the only motivating factor to investigate the health service was as a result of the expose by the Irish broadcaster rather than genuine concern for ensuring that health care standards are met.

#### Accountability and Governance

Tweets reveal immense dissatisfaction with the health care authorities as it was believed that if the recommendations of previous investigations on the Irish Maternity Services had been implemented these perinatal deaths would have been avoided (Textbox 3).

The tweets also revealed concern that there are no obvious implications when recommendations are not implemented or adhered to. Moreover, these tweets revealed how individuals appeared to be resigned to the fact that no official within Government or the health care authorities would be identified and held responsible for these failures (Textbox 3).

Given the belief that neither the Government or the health care authorities would take appropriate action, a criminal investigation was endorsed.

**Examples of tweets showing accountability and governance.**

“Immense Dissatisfaction”

#rtept sounds like complacency was rife in #Portlaoise no action taken on recommendations leading to unnecessary deaths, CTG, oxytocin

How many reviews and investigations do we need to have before change occurs - women and babies and all families deserve better #rtept

“No Implication, No Responsibility”

And tomorrow we Will discover that nobody Will be held accountable! #hse #rtept

The frustrating thing is nothing will change, where's the accountability, where is the governance. #Rtept

yes but we hear that “the hospital” is to blame, convenient to blame a building #noaccountability

James Reilly YOU are the head of the health service and have failed people across the board, change must come from the top down #portlaoise

It's so important that someone is held responsible for the deaths of the babies in Portlaoise Hospital. Can't be allowed to fade from media.

“Criminal Investigation”

Isnt failure to act.....negligence, and when it results in death, manslaughter...? #rtept

@Newstalkfm what was allowed to continue is worse than negligence, it was criminal, the gardai should be sent into that hospital

#### Institutional Responses

Governmental responses, which were shared on Twitter by those in media, initially rallied behind the online reactions describing the events which occurred in Midland Regional Hospital, Portlaoise as inappropriate and unacceptable. The Government made assurances that a thorough investigation would be undertaken and the findings from this investigation would be acted upon to safeguard against similar events happening again (Textbox 4).

Twitter was not utilized as a platform by any health care authority to release a statement in relation to the perinatal deaths. The initial response from medical institutions and professionals from the obstetric community utilized traditional methods of communication to inform the public that the maternity services were safe, making reference to the countries’ rates of perinatal death. These statements were then edited and tweeted by those working within media (Textbox 4).

**Examples of tweets showing government and institutional responses.**

“Governmental Responses”

Emotional Health Minister says Portlaoise concerns will be addressed

Health Minister James Reilly says he was deeply disturbed by the RTE Prime Time revelations about child deaths at Portlaoise Hospital

Kenny: No family should have to fight for truth in our health system (via @thejournal_ie)

Minister Shatter calls the manner in which Portlaoise Hospital treated families, as revealed in recent days, inexcusable.

“Institutional Responses”

The @RCPI_ObsGyn has issued a statement to reassure people about Irish maternity services following last night's #rtept programme.

Prof Fionnuala McAuliffe, @RCPI_ObsGyn, says Ireland is a very safe country in which to have a baby, with low rates of perinatal deaths.

As the public’s concern about the Irish maternity services rose it became more evident that there may be possible implication on service attendance. The Government responded by focusing on reassuring the public that these services were indeed safe.

EK: Portlaoise “will ensure that Ireland will continue to be recognised as one of the safest countries in the world in which to give birth”

Gilmore reassures women over maternity services

#### Unsafe Maternity Services

Concern and uncertainty arose whereby the professional motives of the obstetric community and the Government were called into question. Status updates over the two months indicated the skepticism that was generated in relation to the management of not only the unit in question, which was branded as unsafe, but also the governance of the entire maternity service in Ireland (Textbox 5).

**Examples of tweets showing skepticism and doubts surrounding safety.**

“Skepticism”

Optimum ratio of midwives to patients 1:28. Ratios at Portlaoise hospital 1:75. How can this be possible?#unsafelabours

#portlaoise maternity service cannot be regarded as safe and sustainable within its current governance arrangement

I wonder if all maternity units were looked at would the others come out squeaky clean? So sad for those women and their families #rtept

The claim that Ireland is one of the safest countries in which to give birth ringing ever more hollow #rtept

“Doubts Surrounding the Safety”

HSE probe fifth baby death at Portlaoise Hospital as damning report brands it “unsafe”

I suspect you will be hearing a lot about 'never events' and Portlaoise Hospital in the coming days. CMO report must be read to be believed.

In 2006, 08, 09 & 12, there were 4 neonatal deaths at the maternity unit in Portlaoise from 'never events' according to CMO

Portlaoise report recommends adverse events in low risk pregnancies to be deemed “never events”.

The publication of the investigation by the Chief Medical Officer further reinforced doubts about the safety of Portlaoise hospital. In the report the Chief Medical Officer states that, in Ireland in a low-risk pregnancy, any maternal or perinatal death associated with labor or delivery are to be documented as perinatal “never events”[15].

Tweets indicated that there was a lack of confidence in the quality of care currently being provided, with some women tweeting their reluctance to engage with or attend the services as they did not consider the services safe.

I’d rather give birth on the side of the road than in that hospital #RtePT #HSE heartbreaking stuff

Heartbreaking and chilling at the same time- has unsettled me #expectingno3 #rtept

That won't ease my worries though, due to have baby in Portlaoise in early July :(

As someone due to give birth soon, I'll [be] watching that CTG trace monitor like a hawk after #rtept & demanding fast action if needed.

## Discussion

### Principal Findings

From January 29 till March 31, 2014 there were 3577 status updates; including tweets, retweets and replies, posted on Twitter relating to a cluster of perinatal deaths that occurred in a maternity unit in the Republic of Ireland. Of these status updates, 40% were posted during January 29-31 which coincided with the airing of the current affairs program which brought the perinatal deaths to the attention of the public.

### Limitations

The content of social media can be exploited by health care authorities whereby an analysis of tweets allows health care authorises to identify and respond to concerns [3,13]. However, our study may be limited by a few factors. First, the aim of the study was to evaluate the response on Twitter to the perinatal deaths; however, we restricted our search to publicly available status updates. Twitter is an open forum where it is possible for connections to be nonreciprocal as a person may choose to follow an account and may not be followed in return. Yet, there are exceptions whereby account holders are given the opportunity to protect their account and make status updates available solely to those they give permission to. Twitter also provides the facility for users who are following each other to direct message each other, these messages are private and are not searchable through the search facility on Twitter. Second, our study may be limited by our search terms; however, we believe our search strategy was comprehensive as our list of terms was initially developed independently by two of the researchers (SM and LC) and that all terms identified by both researchers were included in the final list of search terms. The data from this study show that interest in the events spiked and declined quickly which is similar to previous published data; however, it would be of interest to examine the perception of perinatal death over a longer period of time to ascertain any differences in public reaction. Finally, studies have illustrated that Twitter is not fully representative of the general population [25] and therefore the results may be limited by selection bias. It would be of interest to determine if these findings would be observed following the examination of other social networking sites such as Facebook and/or following investigations which utilize more conventional social research methods offline.

### Comparison With Prior Work

The findings of this study support the statement, as reported by Ampofo et al, that people now use digital space to instantaneously evaluate and share their experiences of health care services while, in this instance, watching a national current affairs TV broadcast [10]. By analyzing this content our study identified a number of key themes highlighting the concern about the events which occurred in the hospital but also regarding the governance of the entire health care service. During the course of the television broadcast the tweets indicated that individuals were both shocked and distraught by the events which resulted in four perinatal deaths. Over the course of the broadcast the sentiment observed in the tweets transitioned from distress to anger. Of particular concern to the online community was the manner in which the hospital management were seen to have made attempts to supress information from parents about the events surrounding their babies’ deaths. In the following days, as more details emerged and were shared by the media about the cases, the governance of the maternity services and the health care service as a whole were called into question. Our study identified such a level of dissatisfaction with the governance that a demand for a criminal investigation was called for. Similar to Burnap 2014 [27], our findings illustrate that the frequency of tweet and retweets peaked in line with specific events; initially broadcasting of the television program and subsequently the announcement of an enquiry and the publication of the enquiry.

Research in relation to health communication is now focused on the participatory nature of the Internet with particular reference to social media [13]. This reveals how the public can play a larger role in the various stages of knowledge translation which includes information generation, filtering, and as well as knowledge amplification [13]. Our findings highlight the participatory nature of social media, in particular the filtering and amplification of knowledge generated around the perinatal deaths. This study found that almost half of all status updates which were replies were posted on the 30th of January when the TV episode “fatal failures” was aired. Replies, when individuals were posting a response to a tweet, were indicative of individuals either supporting a statement or sentiment posted or querying the content which was posted. Chou et al state how this process has transformed the pattern of health-related communications, whereby online information sharing is considered more democratic given that it can be controlled by the patients, who share the information of importance to them [28].

However, these developments have raised concerns among health professionals and policy makers [29]. Due to the nature of social media information can be generated and circulated to a wide audience very quickly. Thus, although unintended, Chou et al state that negative health impacts due to misinformation can occur [28]. The findings from this study indicate that the deaths which occurred were perceived as preventable and that any future perinatal deaths should be prevented. Thus, given that the tweeters were becoming increasingly distrusting of the Health Service Executive and the Government, our data would suggest that the users considered the information provided online and through the media as more credible. This finding is in line with those of Coleman et al whereby people, in particular those from a lower socioeconomic status, believed that information posted online by those similar to them was more credible [30]. Peterson et al found that online users’ perception of credibility varied and studies indicate that credibility of online information is linked not only with expertise but also trustworthiness [31]. This is illustrated in our study by the tweets in response to statements from both the obstetric community and the government. Efforts to reassure the women that the maternity services were safe, by making reference to perinatal statistics, were considered deceptive and were believed to be misleading. This perception was reinforced following the publication of the Chief Medical Officer’s report which stated these deaths should from now on be considered as perinatal “never events” and that if such an event were to occur in the future, no reassurance can be derived from summary statistics such as perinatal mortality rates [22].

This study found that one third of all the content generated on Twitter in relation to the perinatal deaths were from media outlet accounts or media personnel. Almost all the content tweeted by media outlets were tweets of original content including news reports and updates. Media personnel were more likely to retweet the information generated by media outlets. This activity is suggestive of the influence the media has within digital spheres and its potential to influence not only perceptions but also patients’ decision making in relation to health care services. Ampofo et al refer to this process as “mediatisation” whereby the logic of the media guides behaviors and decision making throughout society [10]. This phenomenon was also identified in a study undertaken by Donelle and Booth who demonstrated that tweets and public discussions related to health promotion were shaped by a political-media social dynamic [24]. These authors concluded that it is important to determine how the influence of this dynamic on the representation of health, through social media, impacts on the public perception and interaction with health care [24]. Our findings suggest that during this period tweeters perceived the Irish maternity services as unsafe. A recent news article has indicated that there has been a 12% reduction in the number of births in the hospital under review, with the Health Service Executive confirming that in the direct aftermath of the controversy a reduction in attendances at booking clinics was observed [32].

The findings from this, and other similar studies, have shown that Twitter may have the potential to influence patients’ decision making and behavior. Twitter was not utilized as a platform by any health care organization or authority to release a statement in relation to the perinatal deaths. One consequence of this is that the message was broadcasted through the media, which potentially inhibits the ability for the intended message to be delivered to its target audience. Lagan et al stress that health professionals must acknowledge that decision making is influenced by the information which is sought and consumed by patients online [5]. These authors state that there is a need for health professionals to engage in this process and that this engagement would allow them to direct patients to both comprehensive and accurate information. Thackery et al further state that it is important that health care professionals and policy makers engage and exploit the participatory nature of these technological developments [33]. These new e-patients expect interaction and that social media not be used as “virtual pamphlet walls” [24].

### Conclusion

Twitter activity provides a useful insight into attitudes towards health-related events. The role of the media in influencing opinion is well documented and this study underscores the challenges that clinicians face in light of an obstetric media scandal. Given that patients are now likely to access health-related information online, it is imperative that health care providers are meeting the needs of potential service users. Our study highlights the need to exploit social media effectively in order for health care providers and policy makers to identify and respond to the concerns in relation to health care services. Further study to identify how the obstetric community could develop tools to utilize social media sites, such as Twitter, to disseminate valid health information could be beneficial.

## Abbreviations

RTE: Raidió Teilifís Éireann
